# Cortical activation changes and improved motor function in stroke patients after focal spasticity therapy– an interventional study applying repeated fMRI

**DOI:** 10.1186/s12883-015-0306-4

**Published:** 2015-04-11

**Authors:** Ulla Bergfeldt, Tomas Jonsson, Lennart Bergfeldt, Per Julin

**Affiliations:** Division of Rehabilitation Medicine, Department of Clinical Sciences, Danderyd University Hospital, Karolinska Institutet, Stockholm, Sweden; SMILE, Stockholm Medical Imaging Laboratory, Karolinska University Hospital Huddinge, Karolinska Institutet, Stockholm, Sweden; Department of Diagnostic Medical Physics, Karolinska University Hospital Huddinge, Stockholm, Sweden; Department of Molecular & Clinical Medicine/Cardiology, Sahlgrenska Academy, University of Gothenburg, Gothenburg, Sweden; Present address: Center for Advanced Reconstruction of Extremities, Sahlgrenska University Hospital/Moelndal, House U1, 5th floor, SE- 431 80 Moelndal, Sweden

**Keywords:** Stroke, Muscle spasticity, Botulinum toxin, Physical therapy, Magnetic resonance imaging

## Abstract

**Background:**

Impaired dominant hand function in stroke patients is a common clinical problem. Functional improvement after focal spasticity therapy is well documented but knowledge about central correlates is sparse. Brain activity was therefore followed during therapy with repeated functional magnetic resonance imaging (fMRI). The purpose was to analyse motor function and central nervous system (CNS) correlates in response to a standardized motor task in stroke patients after a comprehensive focal spasticity therapy.

**Methods:**

Six consecutive first-time chronic stroke patients [4 women; mean age (SD) 66 (10) years] with right-sided hand paresis and spasticity were studied. Peripheral effects after focal spasticity management including intramuscular botulinum toxin type A (BoNT-A) injections were assessed on 3 occasions (baseline, 6 and 12 weeks) with functional tests. Brain effects were assessed on the same occasions by fMRI blood oxygen level dependent (BOLD) technique during a standardized motor task focusing on the motor and pre-motor cortex (Brodmann areas, BA4a, BA4p & BA6). For reference 10 healthy individuals [5 women; mean age (SD) of 51(8) years], were studied twice with ≥ 6 weeks interval.

**Results:**

After therapy there was a significant reduction in spasticity and functional improvement in 5 of 6 patients. In response to the motor task there was a ~1.5 - 3% increase in brain activity in the motor and pre-motor cortex. At baseline, this increase was larger in the non-injured (ipsilateral) than in the contralateral hemisphere. Compared with healthy subjects the patients showed a significantly (2–4.5 times) higher brain activity, especially on the ipsilateral side. After therapy, there was a larger decrease in the ipsilateral and a minor decrease in the contralateral response, i.e. a clear lateralization of left-to-right in a normalizing direction in all areas.

**Conclusions:**

Comprehensive focal spasticity management was also in this study associated with brain reorganization in a “normalizing” left/right lateralization direction in addition to improved motor function. Furthermore, quantification of BOLD intensity in specified BAs showed reduced neuronal “over-activity” in the injured brain after therapy.

**Electronic supplementary material:**

The online version of this article (doi:10.1186/s12883-015-0306-4) contains supplementary material, which is available to authorized users.

## Background

The adult brain retains a capacity for plasticity and functional reorganization throughout the life span [1]. Stroke is the leading cause of long-term disability in adults. Applying modern imaging techniques, the neural correlates to motor recovery after stroke [2], as well as major differences in cortical activity between stroke patients and healthy controls have been reported exemplifying brain plasticity [2-7]. Because the stroke population is heterogeneous from various perspectives necessitating individualized management, improved understanding of the mechanisms underlying cerebral reorganization and recovery of function is required.

Spasticity occurs in at least 20% of all stroke patients [8,9], contributing to movement deficits, pain and impaired function. Spasticity therapy has therefore become important in today’s neurological clinical rehabilitation [10-12]. Intramuscular injections with botulinum toxin type A (BoNT-A) in combination with physical therapy and orthoses has become an increasingly applied therapeutic alternative and results in functional improvement in up to 90% of patients [13-15]. Intriguingly, in our previous study of 100 patients receiving focal spasticity therapy ~ 30% reported improvements in social participation, linguistic ability and increased cognitive capacity [14]. Furthermore, improved health related quality of life was also seen after focal spasticity therapy in our subsequent study [16]. These observations suggest significant central effects. BoNT-A therapy and subsequent brain modulation has previously been studied, mostly focusing on the extent of neural activation and its changes applying functional magnetic resonance imaging (fMRI) using Blood Oxygenation Level-Dependent (BOLD) contrast [17-21] (see Discussion).

The purpose of this study was to shed light on cortical correlates to peripheral functional improvement after comprehensive focal spasticity therapy in stroke patients studying fMRI changes in intensity and extension of BOLD activity, focusing on the motor and pre-motor cortex.

## Methods

This study was performed in two steps. In the first step the test paradigm, its time-dependent variability and the relation between frequency and brain response were assessed in healthy subjects. In the second step stroke patients were studied.

First, 10 healthy volunteers [5 women; mean age 51 years (SD 8)] were enrolled and fulfilled the following criteria: right-hand dominance, no known acute or chronic disease, and no contraindications to MRI.

Second, 6 first-time chronic stroke patients were studied [4 women; mean age 66 years (SD 10)]. They fulfilled the following inclusion criteria: left-sided stroke ≥ 6 months prior to the study, right-hand dominance, spasticity in the finger flexors of the right hand (score < 4 on the Ashworth Spasticity Scale (AS) of 0–4), partial ability to extend the finger extensors, muscular strength ≥ 2 (Medical Research Council Scale, 0–5), ability to understand and follow instructions and to communicate, no contra-indications for MRI.

Patient characteristics and therapy specifications are shown in Table 1.

**Table 1:** **Patient characteristics (n = 6) at referral, therapeutic targets, and spasticity therapy**

**Patient**	**# 1**	**# 2**	**# 3**	**# 4**	**# 5**	**# 6**
**Sex**	F	F	M	F	M	F
**Age**	67	55	59	79	75	59
**Diagnosis/localization**	Cerebral infarction, left, subcortical	Cerebral infarction, left, subcortical	Cerebral infarction, left, subcortical + brain stem infarction	Cerebral infarction, left, subcortical	Cerebral infarction, left, subcortical	Brain stem + cerebellum
**Years since injury**	2.5	2	2.5	3	1.5	17
**Concomittant diseases/conditions**	Hypertension		Hypertension		Hypertension	
**Medication**	Baclofen, Felodipin, ASA, Dipyridamol	ASA, Dipyridamol, Lanzoprasol	Baclofen, Fenytoin, Clopidogrel, Pravastatin	ASA, Dipyridamol, Bisoprolol	Baclofen, ASA, Diltiazem, Dipyridamol, Amilorid, Hydroklorthiazid, Acetylcystein, Budesonid, Formoterol	Warfarin
**Activity limitations/ICF**	d4400, d4401, d4403	d4400-4403	d4400-4403	d4401-4403	d4400-4403	d4400-4403
**Ashworth baseline wrist/finger/thumb = average**	2/2/1 = 1.7	2/2/2 = 2.0	2/3/1 = 2.0	1/2/1 = 1.3	2/1/1 = 1.3	3/3/2 = 2.7
**Sensory function 2-PD**	3	4	4	3	3	4
**Physical therapy**	-	-	-	-	-	-
**Occupational therapy**	-	x	-	-	-	-
**Orthosis**	-	-	-	-	-	-
**Technical aid**	walking stick, walker	-	wheel chair	walking stick, walker, wheel chair	walking stick, walker	walker
**Therapeutic targets & spasticity therapy**						
**Injected muscle groups**	fl dig superfic & profundus, fl poll long, flex carpi uln & radialis	fl dig superfic & profundus, fl poll long, flex carpi uln & radialis, brachialis, brachio-radialis	fl dig superfic & profundus, fl poll long, flex carpi uln & radialis, pron teres, biceps, brachialis, pectotalis	fl dig superfic & profundus, fl poll long, flex carpi uln & radialis	fl dig superfic & profundus, fl poll long, flex carpi uln & radialis, pron teres, biceps, brachialis	fl dig superfic & profundus, fl poll long, flex carpi uln & radialis, pron teres, biceps, brachialis, pectoralis
**BoNT dose [U]**	120	300	350	130	350	390
**Physical therapy**	x	x	x	x	x	x
**Occupational therapy**	-	x	-	-	-	-
**Orthosis**	x	x	x	x	x	-

This study was conducted according to the principles of the Declaration of Helsinki, and was approved by the Karolinska Institutet Ethics Committee.

### Therapeutic procedure and management in stroke patients

The management strategy has previously been described in detail [14]. Intramuscular injections with BoNT A (Botox® Purified Neurotoxin Complex, Allergan, Inc., Irvine, CA, USA) were administered to finger, wrist and forearm muscles when muscle over-activity was present. Electromyography recording was used to detect abnormal muscle activity and to guide the injections. Dosing was based on the “Guidelines for the use of botulinumtoxin (BTX) for the management of spasticity in adults” [12]. Physiotherapy consisted of supervised hand and wrist training one hour once a week combined with a daily home training program of 45 minutes, and the application of orthoses for flexor muscle stretching; Table 1. The training program emphasized hand function in the hemiparetic hand although motor tasks involving both sides were included. A modified Carr and Shepard motor relearning program for stroke in the upper extremities was applied, also comprising exercises for shoulder abduction, forward flexion, extension and elbow flexion and extension to elicit muscle activity and to train motor control [22]. For the wrist function exercises were mainly aimed at extension, supination and radial deviation. In the hand, training was performed to enhance major hand functions: to grasp, release, and manipulate objects. The program also included exercises with therapeutic dough of varying resistance for dexterity, coordination and strength. Stretching to improve or maintain length of muscles and to prevent contractures ended the program. Patients were also encouraged to involve both hands in everyday tasks.

Treatment was given by one physiotherapist and functional effects were evaluated by another physiotherapist applying tests described below and in an additional file [see Additional file 1].

### Functional tests in stroke patients

Motor function and sensibility were assessed within a week prior to therapy and again 6 and 12 weeks after initiation of therapy and documented by a video recording. The tests are listed in an additional file [see Additional file 1].

### CNS correlates in healthy volunteers and stroke patients

#### Experimental design and procedure

The fMRI protocol was performed twice in healthy subjects with at least 6 weeks interval and three times in patients, at baseline and 6 and 12 weeks after initiation of focal spasticity therapy. A block design was used with 5 repetitions of the motor task lasting 32 s each time. A total of 104 echo-planar imaging (EPI) volume images were acquired. The first 4 volumes (16 s) were excluded to avoid signal saturation effects. The motor task repetitions consisted of 8 volumes each (32 s) of active finger extension-flexion of the right hand in the full range of motion from closed fist to fully open hand with extended fingers at a frequency chosen by each subject. A 12 volumes (48 s) resting phase was inserted at the beginning and end of the run as well as between each activity phase. The entire run therefore lasted altogether ~ 7 min.

Prior to the actual test, the procedure was explained and followed by a training session inside the MR scanner where the subjects listened to pre-recorded instructions via a headset. The same instructions were used in the monitoring room to synchronize fMRI recording and data glove registration. The data glove (5DT, Fifth Dimension Technologies, CA., USA) was used to register the extension-flexion cycle frequency and to monitor compliance with the instructions. This one-size-fits-all data glove made from lycra stretch fabric contains one embedded fiber optic sensor per finger. The optic sensors were linked to the computer via an opto-electronic unit, a ribbon cable, and an interface box. In order to minimize flexor synergies and additional movements in the right wrist, a plastic orthosis was positioned over the wrist under the data glove. Another orthosis was used over the entire left hand and wrist to prevent co-contractions. Head motion was minimized by using a head support system consisting of a deflatable vacuum pillow. During scanning, subjects were blindfolded to reduce eye movements. Headphones dampened scanner noise while a microphone allowed communication between staff and subjects. An E-vitamin tablet was positioned on the right forehead to ascertain right and left in the subsequent analysis.

#### MRI protocol

All measurements were acquired with a Siemens Magnetom Vision 1.5 T whole body MRI system with a standard head coil (Siemens, Erlangen, Germany).

Functional imaging was performed using a T2*-weighted gradient-echo EPI mosaic sequence with TE = 60ms, TR = 4000 ms, FA = 90°, 23 slices covering the whole brain, slice thickness = 5mm, interslice distance = 1mm. The matrix size was 64x64 and field of view (FOV) 240 mm with in-plane resolution 3.75x3.75 mm^2^. Each scanning session included the acquisition of a high resolution TI-weighted 3D scan with a voxel size of 1 × 1 × 1 mm^3^ and a FOV of 256 × 256 mm. A magnetization-prepared rapid acquisition gradient-echo (MP-RAGE) technique was employed. The scan covering the whole brain lasted for 10 minutes and 52 seconds. Furthermore, a T2-weighted and proton density scan was acquired for clinical routine evaluation.

#### Data analysis

Pre-processing and analysis were performed using the fMRI Expert Analysis Tool (FEAT) Version 5.91, which is part of the Analysis Group at the Oxford Centre for Functional MRI of the Brain (FMRIB) Software Library (FSL) (see also www.fmrib.ox.ac.uk/fsl). The following pre-statistics were applied: 1) Motion correction using FMRIB’s Linear Image Registration Tool (MCFLIRT) [23], 2) non-brain removal using the Brain Extraction Tool (BET) [24], 3) spatial smoothing using a Gaussian-shaped kernel with a full width at half maximum (FWHM) of 5 mm, specifying neighborhood size and weighting, 4) grand mean intensity normalization of the entire 4D dataset by a single multiplicative factor, causing all single-session data sets to have the same overall mean intensity, and 5) high-pass temporal filtering (Gaussian-weighted least-squares straight line fitting with sigma = 40.0s) to remove slow unwanted signals (i.e. heart beat, breathing, scanner-related drifts) in each voxel’s time series.

The statistical analysis of fMRI time series was carried out using the FMRIB’s Improved Linear Modeling (FILM) with local auto-correction correlation to fit the GLM voxelwise [25].

In the post-statistics processing of activation images, Z (Gaussianized T/F) statistic images were threshold-using clusters determined by Z > 2.3 and a (corrected) cluster significance threshold of p = 0.05 [26].

Registration to high resolution structural and/or standard space images was carried out using FLIRT, providing optimization for robust affine (12DOF in 3D) linear registration and motion correction of brain images [27].

Atlas based analysis of data was performed using the Jülich probability atlas for the left and right primary and secondary motor cortex-specific Brodmann areas 4a, 4p and 6 to cover primary and secondary motor representations, and Eickhoff’s Anatomy Toolbox v1.5, linearly transformed into MNI152 space [28-30]. For regional probabilistic atlas based analysis parameters were converted to % BOLD signal change. For each probabilistic region of interest (ROI) the activated brain volume (number of adjacent activated voxels) and magnitude of BOLD activity were recorded.

#### Validity, reproducibility & frequency dependence in healthy subjects

The validity of the test paradigm was assessed by the location of the activated voxels. Within the whole brain, the voxel with the highest (peak) BOLD activity was identified by the highest Z-value corresponding to the largest increase in BOLD activity compared with baseline. In addition, the number of all adjacent voxels with a significantly increased BOLD activity was recorded.

The reproducibility was assessed by the distance (mm) in the whole brain between the two voxels with the highest BOLD activity at recording sessions #1 and #2 calculated in space (3D) as well as in the perpendicular X- (frontal), Y- (sagittal) and Z- (vertical) planes. Coefficients of variation (CV; %) were calculated for the peak BOLD activities in paired voxels, where CV was the intra-individual standard deviation divided by the mean of all 20 observations × 100.

Also the numbers of activated voxels were determined within each BA (BA4a, BA4p, BA6) as well as the mean, median, 90% of maximum, and maximum BOLD activity values. CVs were calculated for the number of voxels and BOLD activities for the left hemisphere (L1 vs. L2), right hemisphere (R1 vs. R2), and the left-to-right ratio expressed as (L-R)/R*100 (%). Finally, the BOLD activity was assessed in relation to frequency change in one healthy subject.

The data glove registrations were analyzed with an in-house MATLAB script (The Mathworks, Natick, MA, USA) extracting the finger extension-flexion frequencies for each subject. Frequency-dependence was then tested at the group level at both sessions and in one of the subjects at a third session. In the latter, the same block design was used but instructions were modified by adding the sound of a metronome aiming at one extension-flexion cycle per second (1Hz), one per 2 s (0.5Hz), and one per 4 s (0.25Hz), respectively.

### Statistical analysis

Mean (SD) and median (range) values were used for descriptive purposes. Spearman’s correlation was used for the analysis of the relation between BOLD activity and the motor task frequency. Friedman’s non-parametric test for repeated measures was used for the stroke group analysis. The Wilcoxon’s signed rank test was used for paired comparisons including the post hoc analysis within the stroke group. The Mann–Whitney *U* test was used for comparisons between healthy subjects and patients. A p-value < 0.05 was considered significant. Concerning ROI analyses, however, a conservative p-value using the Bonferroni correction would give p < 0.002.

This was an exploratory study, but, based on previous experience [14], we expected 90% of the patients to be therapy responders.

The software used in the imaging analysis includes statistical methodologies as specified in the specific manuals referred to above.

## Results

### Validity, reproducibility & frequency dependence in healthy subjects

**Figure 1:**
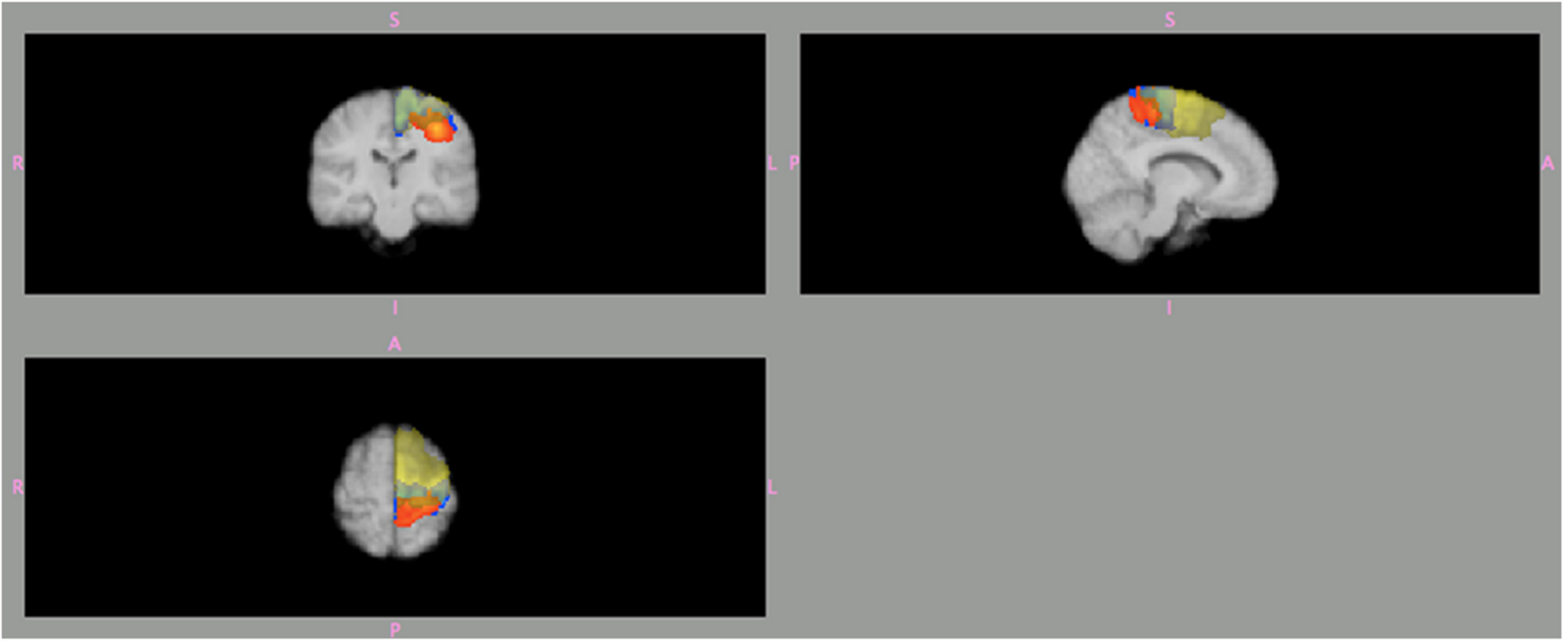
Automatic definition of 3 Brodmann areas (BA) within the left hemisphere according to the Jülich probability atlas BA4a (blue), BA4p (orange) and BA6 (yellow). These areas are projected on to an averaged brain image from the 10 healthy subjects. Note the overlap, which is due to the inter-individual variability of the extension of these 3 areas.

The details of this part of the study are presented in an additional file [see Additional file 1]. In summary, the expected brain areas were activated but the anatomically defined BAs according to the Jülich atlas showed considerable inter-individual overlap; Figure 1. The time-dependent variability was moderate and on the group level there was no relation between the motor-task frequency and the BOLD activity.

### Functional tests in stroke patients

There were 5 (out of 6) therapy responders. Spasticity decreased on average 1.0 from baseline (median 1.85, range 1.3-2.7, vs. 0.85, range 0**-**1.7; p = 0.03, Wilcoxon’s signed rank test). There was a border-line significant improvement in upper extremity function with an average increase of 6.5 points in the total score from baseline (median 21.0, range 5–41, vs. 27.5, range 14–44; p = 0.05, Friedman’s and Wilcoxon’s tests). Specifically, hand function improved in 5 patients, arm function in 4, and wrist function in 2. One patient deteriorated in each function (patient #6). There was no significant change in hand grip strength or sensibility; see Table 2.

**Table 2:** **Functional test results at baseline, and 6 and 12 weeks after therapy initiation**

**Pt#**	**Ashworth scale**			**B Lindmark motor assessment scale**			**Jamar dynamometer**		
	**Average of 3 muscle groups [0–4]**			**Total score [≤63]**			**Mean strength**		
							**Women [~25kg]**		
							**Men [45 kg]**		
	**Baseline**	**6w**	**12 w**	**Baseline**	**6w**	**12w**	**Baseline**	**6w**	**12w**
**1**	1.7	0.7	1.3	19	23	36	1.7	3.7	4.7
**2**	2.0	0.3	1.7	5	5	15	1.7	5	5.3
**3**	2.0	0.7	0.7	11	18	26	11.7	6	7
**4**	1.3	0.3	0.3	41	41	44	3	3.7	4
**5**	1.3	0.3	0.0	25	27	29	6.3	8	5
**6**	2.7	0.7	1.0	21	14	14	6	0.7	1.3
**Median**	1.85	0.5	0.85**	21	20.5	27.5	4.5	4.4	4.9
**Range**	1.3-2.7	0.3-0.7	0-1.7	5-41	5-41	14-44	3-11.7	0.7-8.0	1.3-5.3
**Mean**	1.8	0.5	0.8**	20.3	21.3	27.3*	5.1	4.5	4.6

*p = 0.05; **p < 0.01, Friedman’s test; significant changes for baseline vs. 6 and 12 weeks according to post hoc analysis with Wilcoxon’s signed rank test.

### CNS correlates, stroke patients in comparison with healthy subjects

The overall results in healthy subjects and patients were obtained in the absence of significant changes of motor-task frequency within-groups between the 3 sessions in patients and the 2 sessions in healthy subjects, respectively. There was, however, a significant difference between healthy subjects and patients when comparing the first but not the second sessions, with a lower frequency in patients.

**Figure 2:**
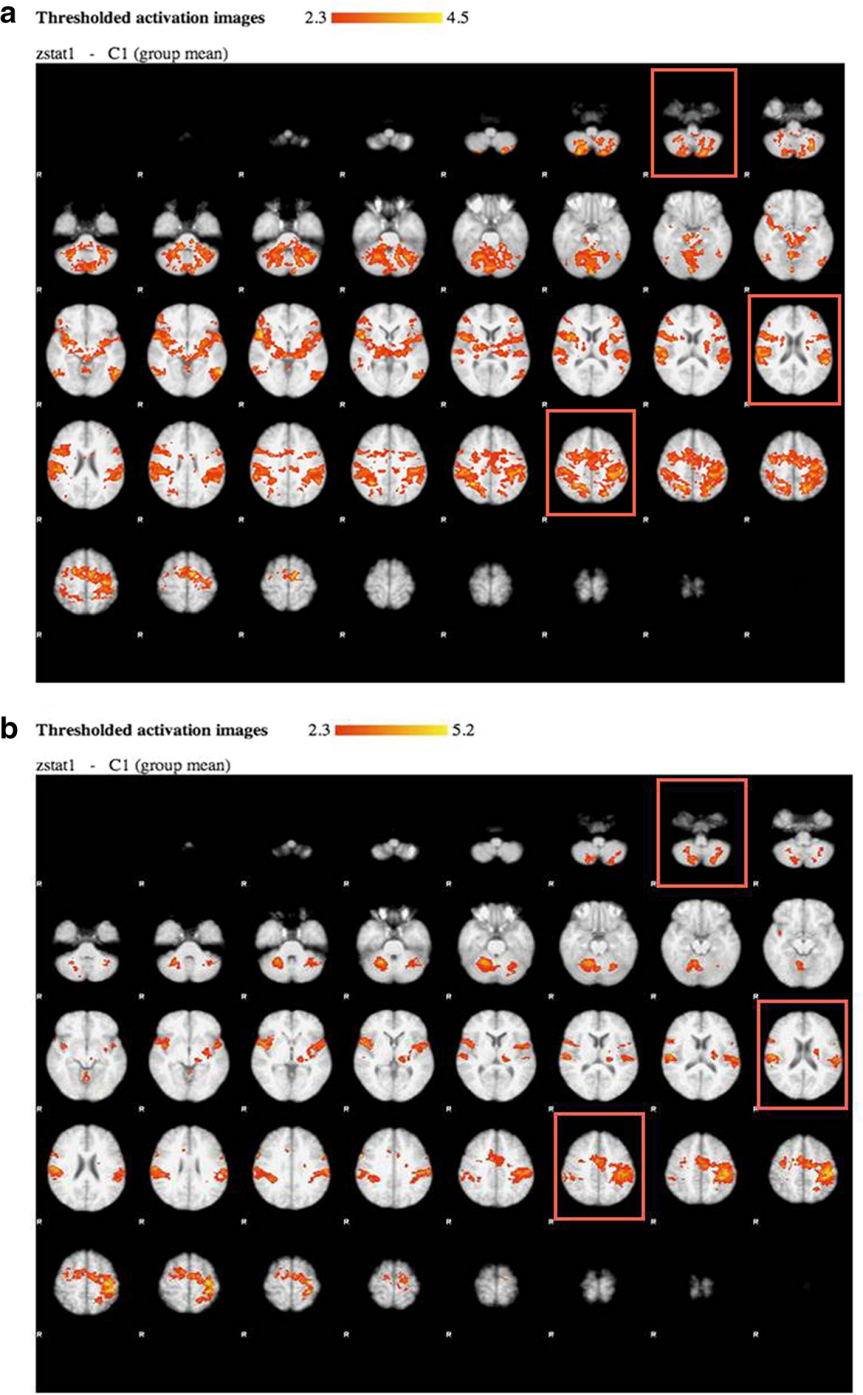
Whole brain statistical analysis of the BOLD activity at a group level during the motor task is shown for 10 healthy subjects at the first **(a)** and the second **(b)** session. Each figure presents the slices of the brain from the base (upper left) to the top (lower right) with a view from below (R = right) with anterior (front) up and posterior (occiput) down. Significantly (Z > 2.3, p < 0.05) activated voxels are overlaid on mean anatomical image. Activation is seen in primary and secondary motor cortices, supplementary motor cortex, and cerebellum, and three brain slice levels in which these areas are visualized are highlighted within coloured rectangles.

The main difference between the two first sessions in patients and healthy subjects is shown in Table 3. It shows that in healthy subjects there is a likely habituation involving the relevant BAs as also illustrated in Figure 2.

**Table 3:** **Changes (%) in mean and maximum BOLD activities in 3 Brodmann areas (BA) during the motor task at session 1 (baseline), 2 (6 weeks), and 3 (12 weeks)**

**BA4a**						
	**BOLD mean**			**BOLD max**		
**Pt #**	**L1/L2/L3**	**R1/R2/R3**	**(L-R)/R %**	**L1/L2/L3**	**R1/R2/R3**	**(L-R)/R %**
1	2.90/2.02/1.86	3.19/0.96/0.95	−9/11/10	19.5/10.1/18.9	22.1/5.52/13.7	−12/84/37
2	2.31/3.52/3.68	2.86/5.58/3.80	−19/-36/-3	10.9/28.4/31.7	16.5/33.5/17.2	−34/-15/85
3	2.24/1.43/3.3	2.67/1.36/2.91	−16/5/13	17.8/10.9/19.7	19.1/8.20/15.0	−7/33/31
4	1.20/2.71/2.18	1.59/2.50/1.82	−25/8/20	8.79/26.8/15.4	13.8/14.9/11.9	−36/80/30
5	2.76/2.64/2.58	1.96/3.38/2.38	41/11/8	22.4/19.8/17.0	18.5/13.7/9.64	21/45/76
6	3.62/0.92/0.71	4.69/1.05/1.28	−23/-12/-45	33.1/3.52/4.35	32.0/7.04/15.1	4/-50/-71
Mean	2.51/2.21/2.39	2.83/2.29/2.19	−8/14/15	18.8/16.6/17.8	20.3/13.8/13.8	−11/29/31
**Healthy subjects; mean**	**1.16/1.05**	0.66/0.49*	**79/130**	**10.6/8.0**	**5.70/3.66**	**108/160**
p values	0.011/0.014	**0.001/0.001**	0.002/0.004	0.034/ns	**0.001/0.001**	0.003**/0.001**
**BA4p**						
	**BOLD mean**			**BOLD max**		
**Pt #**	**L1/L2/L3**	**R1/R2/R3**	**(L-R)/R %**	**L1/L2/L3**	**R1/R2/R3**	**(L-R)/R %**
1	2.37/1.50/1.55	2.42/0.76/0.83	−2/98/87	19.5/10.1/18.9	22.1/4.53/13.7	−12/123/37
2	1.14/2.75/2.50	1.88/3.44/2.77	−39/-20/-10	8.11/28.4/31.7	12.1/22.8/17.2	−33/25/85
3	1.40/0.98/2.65	1.16/0.73/1.44	21/34/84	10.7/7.89/18.1	14.0/5.18/10.8	−24/52/67
4	0.99/1.41/1.26	1.39/1.31/1.30	−29/8/-3	8.79/16.2/6.91	13.8/14.0/11.9	−36/16/-42
5	2.27/2.12/1.45	1.59/1.70/2.18	43/24/-33	18.5/13.9/8.91	18.5/1.1/9.22	0/15/-3
6	2.96/0.86/0.61	2.96/0.70/0.67	0/22/-9	33.1/2.96/4.35	25.6/7.04/5.38	29/-58/-19
Mean	1.86/1.60/1.67	1.90/1.44/1.53	−1/28/19	16.4/13.3/14.8	17.7/10.9/11.4	−13/29/21
**Healthy subjects; mean**	**0.94/0.87**	0.51/0.37*	**85/57**	**8.08/6.73**	**4.16/2.86**	**122/149**
p values	0.045/0.020	**0.001/0.001**	0.003/0.004	0.045/ns	**0.001/0.001**	0.002/0.008
**BA6**						
	**BOLD mean**			**BOLD max**		
Pt #	**L1/L2/L3**	**R1/R2/R3**	**(L-R)/R %**	**L1/L2/L3**	**R1/R2/R3**	**(L-R)/R %**
1	2.81/1.96/1.71	3.10/0.94/0.67	−9/108/155	36.4/13.0/18.7	34.7/13.0/3.56	5/0/430
2	2.59/3.93/3.89	2.64/6.15/4.11	−1/-36/-5	10.9/32.8/33.2	16.5/39.5/28.3	−34/-17/17
3	3.08/1.78/3.81	2.71/1.50/3.59	14/0/619	18.3/13.8/22.7	19.1/9.60/20.4	−4/44/11
4	1.51/1.26/2.62	1.30/2.28/1.98	16/-40/33	15.6/26.8/16.7	8.50/13.6/11.9	84/96/41
5	2.52/3.09/4.72	1.53/2.25/3.64	65/37/30	31.9/24.1/39.0	17.7/14.8/25.1	80/63/55
6	5.09/1.23/0.88	4.10/1.06/1.16	24/15/-24	27.0/13.9/5.99	32.0/10.3/15.1	−16/35/-60
Mean	2.93/2.21/2.94	2.56/2.37/2.52	18/16/32	23.3/20.7/22.7	21.4/16.8/17.4	19/37/82
**Healthy subjects; mean**	**1.09/0.94**	**0.69/0.56**	**61/76**	**12.7/9.62**	**6.08/4.69**	**119/116**
p values	0.003/0.004	**0.001/0.001**	0.015/0.034	0.034/0.011	0.003**/0.001**	0.020/0.015

Values from the 10 healthy subjects studied twice serve as reference; p values refer to between-group differences. *p < 0.05 for session 1 vs. 2 in healthy subjects (p < 0.002 applying Bonferroni correction in bold font).

**Figure 3:**
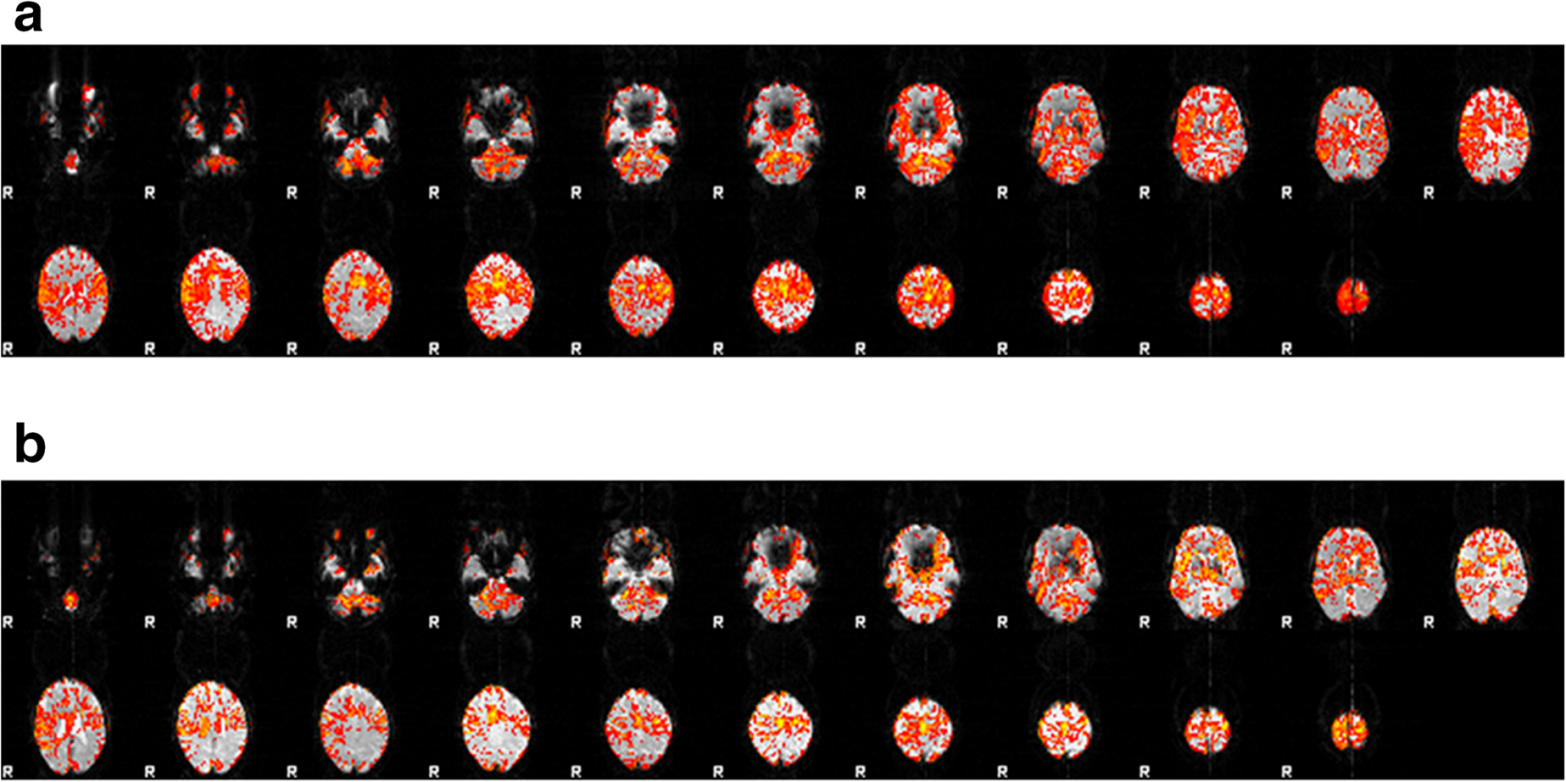
The mean BOLD activity is shown before **(a)** and 12 weeks after **(b)** intervention in stroke patient #1. Each panel presents the slices of the brain from the base (upper left) to the top (lower right) with a view from below (R = right) with anterior (front) up and posterior (occiput) down. Before therapy the BOLD activity is more pronounced than after, especially in the right hemisphere.

In patients, the increase in mean BOLD activity during finger extension-flexion compared to rest was 2-3% in BA4a and BA6 and < 2% in BA4p in the left and right hemispheres both before and after intervention. Compared to healthy subjects, the motor task was associated with a 2–4.5 times greater increase in BOLD activity in patients. The difference in activities between the left and right hemisphere is presented as percentages of the right-sided activity [(L-R)/R*100], where negative values (only in patients) signifies a higher activity in the right hemisphere. In BA4a and BA4p patients had a higher right-sided activity, which changed in a normalizing direction after therapy, exemplified by patient #1 in Figure 3. In BA6 patients showed a more normal left-to-right lateralization throughout the study, but with less difference between the left and right hemispheres than the healthy subjects.

Before therapy, the number of voxels reaching the activity threshold (Z > 2.3), i.e. the extension of significant activity increase, in all 3 areas was on average greater in the left than in the right hemisphere, but there were inter-individual differences. After therapy the distribution of activated voxels was reduced in both hemispheres, most obvious on the right side as exemplified in Figure 3. These changes were generally consistent between patients. Comparing patients vs. healthy subjects there was no significant difference in the number of activated voxels in the left hemisphere. In contrast, there was a larger number of activated voxels in the right hemisphere in patients, significant in all 3 areas in the second session (Mann–Whitney *U*-test). The left-to-right lateralization was significantly more pronounced in healthy subjects than in patients in BA4a and BA4p but not in BA6. In response to therapy, the left-to-right ratios increased in patients but remained less pronounced than in healthy subjects.

## Discussion

The main findings of this study on the central correlates to functional motor improvement in 5 out of 6 first-time hemi-paretic stroke patients with disabling right hand spasticity receiving comprehensive focal spasticity therapy were as follows: Repeated fMRI during a standardized motor task before intervention showed an increase in BOLD activity of on average ~1.5-3% in the motor and pre-motor cortex (BA4a, BA4p & BA6), and a larger increase in BOLD activity in the ipsilateral (right) than in the contralateral (left) hemisphere in BA4a and B4p. After intervention there was a minor decrease in the left-sided and a larger decrease in the right-sided BOLD activity (non-injured part of the brain), leading to a clear lateralization (left > right) in a “normalizing” direction. Compared to 10 healthy subjects, the mean increase in BOLD activity during the motor task was 2–4.5 times larger in patients and with less left-sided dominance. The reduction in BOLD activity (intensity) during the motor task performed after therapy likely reflects reduced oxygen and nutrient requirements.

While the peripheral mechanism of BoNT-A treatment is well-known, knowledge about CNS effects is sparse. Recently, evidence for central effects following focal spasticity therapy with BoNT-A assessed both with real and imaginary finger movements, have, however, been presented. fMRI was previously mainly applied to follow changes in the extension (areas) of brain activation. One explanation offered for the observed changes in activation pattern is alterations in sensory inputs to the CNS following reduction of Ia afferent signals [19-21]. These observations are in line with results from studies on focal dystonia describing central effects of BoNT-A treatment [31-33]. Like in previous studies [17-21], a whole brain approach was initially applied also in our study first using the Brain Voyager software showing main activities in the motor and pre-motor cortex (data not presented). A more automatic and less observer dependent method became available and was therefore applied. In the fMRI analysis (with the Oxford FMRIB Software Library, FSL), after whole brain analysis (see Additional file 1 Tables S1A and B), we focused on quantifying BOLD intensity in motor cortex-specific BAs using the Jülich probability atlas [29,30]. Apart from confirming previous observations on brain reorganization following focal spasticity therapy, this study showed that the same motor task involved more intense brain activation in stroke patients than in healthy subjects, especially in the ipsilateral (non-injured) hemisphere, and that this activation was significantly reduced following therapy. Tentatively, these changes might contribute to reduced fatigue and improved social and cognitive function observed in our previous studies (see next section for possible mechanism), an interpretation that, however, needs further study. Please observe that these inferences are drawn from statistical analyses primarily of specific motor areas.

### Comparison of the BOLD activity response in patients vs. healthy subjects

Neural correlates to motor recovery after stroke as well as major differences in cortical activity between stroke patients and healthy controls have previously been reported in several studies applying fMRI [3-7]. In the present study there was a clear difference between patients and healthy subjects with regard to the intensity and extension of the BOLD activity. The difference was most significant concerning the activation of the right hemisphere. In healthy subjects the BOLD activity became even less at the 2^nd^ session – likely a habituation effect observed before [34]. A decrease in BOLD activity was also observed in patients after therapy, especially in the right hemisphere. Even if part of this decrease was due to habituation, the pattern was different than in healthy subjects.

The intriguing issue regarding the differences between patients and healthy subjects concerns the complex relation between BOLD activity, cerebral blood flow (CBF) and oxygen utilization or metabolism (cerebral metabolic rate of oxygen, CMRO_2_): *Does the stroke brain consume more energy even for a simple motor task?* Unfortunately, and for technical reasons, absolute CBF could not be measured in this study – a limitation. In a study of healthy subjects performing self-paced bilateral finger tapping, a linear relation between increased CBF and CMRO_2_ with a ratio of 3:1 was reported, but the relation between BOLD activity and CBF or CMRO_2_ was not addressed [35]. Based on subsequent seminal work from San Diego [34,36,37] some assumptions can be made. A reduction in BOLD activity in response to a standardized task might be due to reduced neuronal activity coupled to reduced CMRO_2_ and CBF [37]. However, in stroke patients the hemodynamic response to neuronal activity might be altered. In healthy subjects the ratio between CBF and CMRO_2_ had a good reproducibility (CV < 10%) at least in visual experiments [34]. This suggests that most of the decrease in BOLD activity we observed in our healthy subjects was due to a decrease in neuronal activity (by habituation), leading to a net decrease in oxygen consumption and CBF. Already at baseline our stroke patients had a higher BOLD activity especially in the right non-injured hemisphere. In a previous study the CBF was higher in the right than in the left hemisphere and similar to age-matched healthy subjects, but less than in younger healthy subjects [38]. Therefore at least part of the difference in BOLD activity between healthy subjects and patients at the first session in our study might be explained by an age-related CBF difference. The reduction of BOLD activity in the right hemisphere in a “normalizing” direction after therapy might be due to reduced neuronal activity (“over activity” of the non-injured hemisphere) during the motor task, but could also be related to changes in the vascular response or the CMR0_2_ and could be dependent on the baseline absolute CBF of the region. This issue deserves further detailed study.

### Focal spasticity therapy

In patients with upper motor neuron syndrome (UMNS), spasticity may cause disability when it leads to structural shortening of muscles, weakness and pain, contributing to movement deficits and impaired function. In our study, with the therapeutic approach to minimize or eliminate the consequences of spasticity, we investigated the effects of a comprehensive spasticity management regarding peripheral as well as central aspects. Before therapy, the average spasticity score ranged between 1.3 and 2.7 (Ashworth scale), a moderate muscle over-activity. Spasticity was assessed at rest, but is often observed to increase during activity. Muscle over activity caused impaired hand and finger dexterity and speed in all patients, but decreased significantly after therapy with an average of 1.0, in concordance with previous reports [13,14,39].

Improvements in stroke patients after rehabilitation using BoNT-A with or without physiotherapy, have been shown in previous studies, also reporting on changes in central correlates during a motor task detected with fMRI [17-21]. These studies used similar methods, task and study plan as in this study. All of them applied BoNT-A to reduce hand spasticity but only three articles reported on combining BoNT-A and intensive physiotherapy [19-21], which is recommended for focal spasticity treatment [12,40,41]. In another article patients performed repetitive arm cycling following BoNT-A injections [17], and in yet another study no physiotherapy was performed, except for stretching [18]. All recent studies confirm and describe the patterns of cortical reorganization following therapy in chronic stroke patients in line with previous studies showing that training modulates cortical reorganization [42-44]. Some uniformity is thus emerging concerning improved motor function and brain reorganization and general principles for therapeutic strategies. However, patient heterogeneity predicts that the same therapeutic approach will not be appropriate for all individuals. Therefore further studies on the effect of different physiotherapy or training strategies in combination with BoNT-A therapy are needed.

### Study limitations

We planned to include 10 stroke patients, but did not reach this goal before new equipment was installed in the MR-lab. Despite the limited number of patients the results were consistent in the group of responders, which also represent the proportion expected from previous studies [13,14]. Another limitation mentioned before is that absolute CBF, such as ASL (arterial spin labeling) MRI could not be assessed with the technology available at the time of the study. Proofs of a relation between improved motor function, positive side-effects, and a brain correlate have not been provided in this study. In this first step, we focused on improved motor function, its brain correlate and changes in BOLD activity. Further research is needed including assessment of e.g. fatigue, its changes after therapy and the changes in brain response. Finally, the healthy subjects were not enrolled to match the patients, but rather to serve as a reference of normal physiology when using the test paradigm and protocol.

## Conclusions

Comprehensive focal spasticity management was also in this study associated with brain reorganization in a “normalizing” left/right lateralization direction in addition to improved motor function. Furthermore, quantification of BOLD intensity in specified BAs showed reduced neuronal “over-activity” in the injured brain after therapy.

## Additional file

Additional file 1:
**Part 1: Functional tests - patients.** Part 2: Validity, reproducibility & frequency dependence in healthy subjects. **Table S1A** Peak activities expressed as Z-values at each recording session (#1 & #2; Z >2.3 means p<0.05). **Table S1B.** The coordinates for each peak activity voxel and their corresponding Brodmann areas according to the Jülich probability atlas (Eickhoff et al., [29,31]) at each session (#1 & 2). **Table S2.** Individual data and coefficients of variation in healthy subjects for the mean BOLD activities expressed as percent increase compared with baseline. **Table S3.** Individual data and coefficients of variation in healthy subjects for the maximum BOLD activities expressed as percent increase compared with baseline. **Table S4.** fMRI changes in session #1, 2, and 3, in one healthy individual (#1) performing a motor task consisting of 5 repetitions of 30s active finger extension – flexion of the right hand in the full range of motion at a frequency freely chosen (#1 and 2) and metronome guided (#3).

## Abbreviations

BA: Brodmann area
BOLD: Blood oxygen level dependent
BoNT- A: Botulinum toxin type A
CBF: Cerebral blood flow
CMRO_2_: Cerebral metabolic rate of oxygen
CNS: Central nervous system
fMRI: Functional magnetic resonance imaging
UMNS: Upper motor neuron syndrome

## References

[1] Johansson BB (2003). Neurorehabilitation and brain plasticity. J Rehabil Med.

[2] Ward NS, Brown MM, Thompson AJ, Frackowiak RS (2003). Neural correlates of motor recovery after stroke: a longitudinal fMRI study. Brain.

[3] Pineiro R, Pendlebury S, Johansen-Berg H, Matthews PM (2001). Functional MRI detects posterior shifts in primary sensorimotor cortex activation after stroke: evidence of local adaptive reorganization. Stroke.

[4] Nudo RJ, Plautz EJ, Frost SB (2001). Role of adaptive plasticity in recovery of function after damage to motor cortex. Muscle Nerve.

[5] Fraser C, Power M, Hamdy D (2002). Driving plasticity in human adult motor cortex is associated with improved motor function after brain injury. Neuron.

[6] Ward NS, Brown MM, Thompson AJ, Frackowiak RS (2003). Neural correlates of outcome after stroke: a cross-sectional fMRI study. Brain.

[7] Ward NS (2004). Functional reorganization of the cerebral motor system after stroke. Curr Opin Neurol.

[8] Sommerfeld DK, Eek EU, Svensson AK, Holmqvist LW, von Arbin MH (2004). Spasticity after stroke: its occurrence and association with motor impairments and activity limitations. Stroke.

[9] Watkins C, Leathley MJ, Gregson JM, Moore AP, Smith TL, Sharma AK (2002). Prevalence of spasticity post stroke. Clin Rehabil.

[10] Ward AB, Ko Ko C, Barnes MP, Johnson GR (2005). Pharmacological management of Spasticity. Upper motor neurone syndrome and spasticity: clinical management and neurophysiology.

[11] Gracies JM, Elovic E, McGuire J, Simpson DM, Mayer NH, Simpson DM (2002). Traditional pharmacological treatments for spasticity. Part I: Local treatments. Spasticity: Etiology, evaluation, management and the role of botulinum toxin.

[12] Turner-Stokes L, Ward A (2002). Guidelines for the use of botulinumtoxin (BTX) in the management of Spasticity in adults.

[13] Brashear A, Gordon MF, Elovic E, Kassicieh VD, Marciniak C, Do M (2002). Intramuscular injection of botulinum toxin for the treatment of wrist and finger spasticity after a stroke. N Engl J Med.

[14] Bergfeldt U, Borg K, Kullander K, Julin P (2006). Focal spasticity therapy with botulinum toxin: effects on functions, ADL, and pain in 100 adult patients. J Rehab Med.

[15] Simpson DM, Gracies JM, Yablon SA, Barbano R, Brashear A (2009). Botulinum neurotoxin versus tizanidine in upper limb spasticity: a placebo-controlled study. J Neurol Neurosurg Psychiatry.

[16] Bergfeldt U, Sköld C, Julin P (2009). Short form 36 assessed health- related quality of life after focal spasticity therapy. J Rehabil Med.

[17] Diserens K, Ruegg D, Kleiser R, Hyde S, Perret N, Vuadens P (2010). Effect of repetitive arm cycling following botulinum toxin injection for poststroke spasticity: evidence from fMRI. Neurorehabil Neural Repair.

[18] Manganotti P, Acler M, Formaggio E, Avesani M, Milanese F, Baraldo A (2010). Changes in cerebral activity after decreased upper-limb hypertonus: an EMG-fMRI study. Magn Reson Imaging.

[19] Senkárová Z, Hlustík P, Otruba P, Herzig R, Kanovský P (2010). Modulation of cortical activity in patients suffering from upper arm spasticity following stroke and treated with botulinum toxin A: an fMRI study. J Neuroimaging.

[20] Veverka T, Hluštík P, Tomášová Z, Hok P, Otruba P, Král M (2012). BoNT-A related changes of cortical activity in patients suffering from severe hand paralysis with arm spasticity following ischemic stroke. J Neurol Sci.

[21] Tomasova Z, Hlustık P, Kral M, Otruba P, Herzig R, Krobot A (2013). Cortical Activation Changes in Patients Suffering from Post-Stroke Arm Spasticity and Treated with Botulinum Toxin A. J Neuroimaging.

[22] Carr JH, Shepard RB (1996). A motor relearning programme for stroke.

[23] Jenkinson M, Bannister P, Brady M, Smith S (2002). Improved optimization for the robust and accurate linear registration and motion correction of brain images. Neuroimage.

[24] Smith S (2002). Fast robust automated brain extraction. Hum Brain Mapp.

[25] Woolrich MW, Ripley BD, Brady M, Smith SM (2001). Temporal autocorrelation in unvaried linear modeling of FMRI data. Neuroimage.

[26] Worsley KJ, Jezzard P, Matthews PM, Smith SM (2001). Statistical analysis of activation images. Functional MRI: An introduction to methods.

[27] Jenkinson M, Smith S (2001). A global optimisation method for robust affine registration of brain images. Med Image Anal.

[28] Eickhoff SB, Stephan KE, Mohlberg H, Grefkes C, Fink GR, Amunts K (2005). A new SPM toolbox for combining probabilistic cytoarchitectonic maps and functional imaging data. Neuroimage.

[29] Toga AW, Thompson PM, Mori S, Amunts K, Zilles K (2006). Towards multimodal atlases of the human brain. Nat Rev Neurosci.

[30] Eickhoff SB, Paus T, Caspers S, Grosbras MH, Evans AC, Zilles K (2007). Assignment of functional activations to probabilistic cytoarchitectonic areas revisited. Neuroimage.

[31] Gelb DJ, Yoshimura DM, Olney RK, Lowenstein DH, Aminoff MJ (1991). Change in pattern of muscle activity following botulinum toxin injections for torticollis. Ann Neurol.

[32] Ceballos-Baumann AO, Sheean G, Passingham RE, Marsden CD, Brooks DJ (1997). Botulinum toxin does not reverse the cortical dysfunction associated with writer’s cramp. A PET study. Brain.

[33] Opavský R, Hluštík P, Otruba P, Kaňovský P (2011). Sensorimotor network in cervical dystonia and the effect of botulinum toxin treatment: a functional MRI study. J Neurol Sci.

[34] Leontiev O, Buxton RB (2007). Reproducibility of BOLD, perfusion, and CMRO2 measurements with calibrated-BOLD fMRI. Neuroimage.

[35] Kastrup A, Krüger G, Neumann-Haefelin T, Glover GH, Moseley ME (2002). Changes of cerebral blood flow, oxygenation, and oxidative metabolism during graded motor activation. Neuroimage.

[36] Leontiev O, Dubowitz DJ, Buxton RB (2007). CBF/CMRO2 coupling measured with calibrated BOLD fMRI: Sources of bias. Neuroimage.

[37] Perthen JE, Lansing AE, Liau J, Liu TT, Buxton RB (2008). Caffeine-induced uncoupling of cerebral blood flow and oxygen metabolism: a calibrated BOLD fMRI study. Neuroimage.

[38] Brumm KP, Perthen JE, Liu TT, Haist F, Ayalon L, Love T (2010). An arterial spin labeling investigation of cerebral blood flow deficits in chronic stroke survivors. Neuroimage.

[39] Pierson SH, Katz DI, Tarsy D (1996). Botulinum toxin A in the treatment of spasticity: functional implications and patient selection. Arch Phys Med Rehabil.

[40] Hesse S, Werner C (2003). Poststroke motor dysfunction and spasticity: novel pharmacological and physical treatment strategies. CNS Drugs.

[41] Ward AB, Aguilar M, De Beyl Z, Gedin S, Kanovsky P, Molteni F (2004). Use of botulinum toxin type A in management of adult spasticity. A European consensus statement. Eura Medicophys.

[42] Johansson BB (2000). Brain plasticity and stroke rehabilitation. Stroke.

[43] Nelles G (2004). Cortical reorganization-effects of intensive therapy. Restor Neurol Neurosci.

[44] Karni A, Meyer G, Jezzard P, Adams MM, Turner R, Ungerleider LG (1995). Functional MRI evidence for adult motor cortex plasticity during motor skill learning. Nature.

