# Supplementary material for: Cortical activation changes and improved motor function in stroke patients after focal spasticity therapy– an interventional study applying repeated fMRI
Source: BMC Neurol. 2015 Apr 11;15:52. doi: 10.1186/s12883-015-0306-4 (PMC4450484; doi:10.1186/s12883-015-0306-4)
Supplement: Additional file 1: — Part 1: Functional tests - patients. Part 2: Validity, reproducibility & frequency dependence in healthy subjects. Table S1A Peak activities expressed as Z-values at each recording session (#1 & #2; Z >2.3 means p<0.05). Table S1B. The coordinates for each peak activity voxel and their corresponding Brodmann areas according to the Jülich probability atlas (Eickhoff et al., [29,31]) at each session (#1 & 2). Table S2. Individual data and coefficients of variation in healthy subjects for the mean BOLD activities expressed as percent increase compared with baseline. Table S3. Individual data and coefficients of variation in healthy subjects for the maximum BOLD activities expressed as percent increase compared with baseline. Table S4. fMRI changes in session #1, 2, and 3, in one healthy individual (#1) performing a motor task consisting of 5 repetitions of 30s active finger extension – flexion of the right hand in the full range of motion at a frequency freely chosen (#1 and 2) and metronome guided (#3). [file 12883_2015_306_MOESM1_ESM.doc]

Additional file part 1.

Functional tests - patients

1) The Ashworth Scale. Muscle tone was assessed from the degree of resistance to passive movement of the target muscle group and estimated according to the Ashworth Scale, 0-4, where 4 means rigidity [1]. Special attention was paid to optimize the testing position and to control the speed of passive stretching and range of movement. An average muscle tone was calculated for agonistic muscle groups.

2) The Birgitta Lindmark Motor Assessment Scale (BL Scale) was used to assess arm-hand mobility and function [2]. The BL Scale is a modified version of the Fugl-Meyer Sensorimotor Assessment [3] and considered reliable, valid and sensitive [4, 5]. The 2 parts (out of 7, A-G), which concern the upper extremity were used, where A (I=arm, II=wrist, and III=hand) define the ability to perform active movements, and B the velocity of motion. Each item within these parts was evaluated on a 4-point scale (0-3), with a higher score representing better function; a total score was calculated.

3) The Jamar dynamometer (Asimow Engineering Co, Los Angeles, CA 90 024, USA)

was used to measure isometric hand grip strength. A high instrument validity and reliability has been reported [6]. The mean value of 3 contractions was calculated.

4) Two-point discrimination (2-PD) was used to assess sensory function [7].

Additional file part 1. References

1. Ashworth B: **Preliminary trial of carisoprodol in multiple sclerosis**. *Practitioner* 1964, **192**:540-542.

2. Lindmark B: **Evaluation of functional capacity after stroke with special emphasis on motor function and activities of daily living**. *Scand J Rehabil Med* 1988, **21**:1–40.

3. Fugl-Meyer AR, Jääskö L, Leyman I, Olsson S, Steglind S: [**The post-stroke hemiplegic patient. A method for evaluation of physical performance.**](http://www.ncbi.nlm.nih.gov/pubmed/1135616?ordinalpos=5&itool=EntrezSystem2.PEntrez.Pubmed.Pubmed_ResultsPanel.Pubmed_DefaultReportPanel.Pubmed_RVDocSum) *Scand J Rehabil Med* 1975, **7**:13-31.

4. Lindmark B, Hamrin E: **Evaluation of functional capacity after stroke as a basis for active intervention: presentation of a modified chart for motor capacity assessment and its reliability**. *Scand J Rehabil Med* 1988a, **20**:103–109.

5. Lindmark B, Hamrin E: **Evaluation of functional capacity after stroke as a basis for active intervention: validation of a modified chart for motor capacity assessment**. *Scand J Rehabil Med* 1988b, **20**:111–115.

6. Mathiowetz V, Weber K, Volland G, Kashman N: [**Reliability and validity of grip and pinch strength evaluations.**](http://www.ncbi.nlm.nih.gov/pubmed/6715829?ordinalpos=3&itool=EntrezSystem2.PEntrez.Pubmed.Pubmed_ResultsPanel.Pubmed_DefaultReportPanel.Pubmed_RVDocSum) *J Hand Surg* [Am] 1984, **9**:222-226.

7. Bell-Krotoski J, Weinstein S, Weinstein C: [**Testing sensibility, including touch-pressure, two-point discrimination, point localization, and vibration.**](http://www.ncbi.nlm.nih.gov/pubmed/8393725?ordinalpos=2&itool=EntrezSystem2.PEntrez.Pubmed.Pubmed_ResultsPanel.Pubmed_DefaultReportPanel.Pubmed_RVDocSum) *J Hand Ther* 1993, **6**:114-123.

Additional file part 2.

**Validity, reproducibility & frequency dependence in healthy subjects**

The two recording sessions were performed on average 42 days apart. The number of extension-flexion cycles during the 5 sequences of 32 s activities averaged 13 (range 6-21), corresponding to frequencies between 0.19 and 0.71Hz, which did not differ significantly between the two sessions (coefficient of variation, CV 20%). The “mean displacement” (motion correction) was 0.14 mm (SD 0.12) for all 20 sessions, varying between 0.05 and 0.52 mm. There was no significant difference in motion correction between the two sessions.

The CNS (whole brain) correlate to the motor task was found within the expected parts of the brain confirming the validity of the test paradigm, but with considerable overlap between the Brodmann areas (BA); main text see Figure 1; Additional tables 1A & B. The maximum BOLD values in a single voxel did not differ significantly between recording session #1 and #2.

The distance between the 2 voxels with peak BOLD activities at the two sessions was relatively short (3-8 mm) in 5 individuals, whereas the remaining 5 subjects showed larger variations. The median distance between these voxels in space (3D) was 9.9 mm (3.0-58.7), in the frontal plane (X) 6.5 mm (0.1-30.6), in the sagittal plane (Y) 1.3 mm (0.3-28.9) and in the vertical plane (Z) 4.3 mm (0.6-48.8). The between-voxel distances were significantly shorter in all 3 planes compared with in space (p-values < 0.01), but did not differ between the 3 planes. There was a moderate variation in peak activities with a CV of 29%.

During the motor task the mean and maximum BOLD activities in the left hemisphere increased around 1 and 9% in all 3 BAs compared to baseline, respectively. The corresponding figures in the right hemisphere were around 0.5 and 5%, respectively. There were significant differences between BOLD mean values at the two sessions in the following comparisons: BA4a: right hemisphere 1 vs. 2 (p=0.047); BA4p: right hemisphere 1 vs. 2 (p=0.047) and left-to-right ratio 1 vs. 2 (p=0.028). The time-dependent variability of the mean, median, and the 90% of maximum values of BOLD activity was similar in these 3 areas, but generally larger for the maximum BOLD activities. This pattern was consistent for the comparisons within the left hemisphere session 1 vs. 2 and within the right hemisphere session 1 vs. 2; main text figure 2 and 3, Additional file part 2, tables 2 and 3.

At the second session the number of voxels reaching the threshold for significant change in BOLD activity decreased significantly, especially in the right hemisphere and in BA4a and BA4p. As a consequence the left-to right ratios became accentuated at a group and individual level, respectively.

On the group level there was no correlation between the extent or magnitude of BOLD activity on the one hand and the extension-flexion cycle frequency on the other at self-paced flexion-extension (range 0.19 -0.71 Hz). However, in the single subject performing paced extension-flexion at 0.25, 0.5, and 1 Hz, there was an increase in fMRI activity with increasing frequency; see Additional file part 2, table 4.

**Additional file part 2. Table 1A.** Peak activities expressed as Z-values at each recording session (#1 & #2; Z >2.3 means p<0.05). The distance (mm) between the two peak activity voxels is presented in space (3-D) and for each direction (X=frontal plane, Y=sagittal plane, Z=vertical plane).

| **Subj.** | **Z-value** | | **Peak dist. [mm]** | | | |
| --- | --- | --- | --- | --- | --- | --- |
|  | #1 | #2 | 3-D | X | Y | Z |
| **1** | 15.5 | 9.8 | 5.5 | 2.7 | 0.6 | 4.8 |
| **2** | 4.7 | 7.0 | 58.7 | 30.6 | 11.3 | 48.8 |
| **3** | 5.7 | 12.1 | 5.3 | 5.1 | 1.2 | 0.6 |
| **4** | 11.9 | 10.5 | 12.7 | 9.0 | 8.1 | 3.8 |
| **5** | 14.4 | 13.1 | 3.3 | 0.1 | 0.3 | 3.3 |
| **6** | 12.9 | 8.6 | 11.8 | 4.2 | 1.1 | 11.0 |
| **7** | 13.5 | 7.6 | 3.0 | 0.3 | 1.4 | 2.6 |
| **8** | 14.3 | 13.4 | 31.0 | 9.6 | 28.9 | 5.6 |
| **9** | 14.7 | 8.1 | 7.9 | 7.8 | 0.6 | 0.9 |
| **10** | 4.7 | 6.9 | 28.7 | 14.6 | 24.1 | 5.7 |
| Mean | 11.2 | 9.7 | 16.8 | 8.4 | 7.8 | 8.7 |
| SD | 4.4 | 2.5 | 17.8 | 9.0 | 10.6 | 14.4 |
| Median | 13.2 | 9.2 | 9.9 | 6.5 | 1.3 | 4.3 |
| Range | 4.7-15.5 | 6.9-13.4 | 3.0-58.7 | 0.1-30.6 | 0.3-28.9 | 0.6-48.8 |

**Additional file part 2. Table 1B.** The coordinates for each peak activity voxel and their corresponding Brodmann areas according to the Jülich probability atlas (Eickhoff et al., 2005 & 2007) at each session (#1 & 2).

| **Subj.** | **Voxel coordinates [mm]** | | | | | | **Brodmann areas** | |
| --- | --- | --- | --- | --- | --- | --- | --- | --- |
|  | **#1**  **X Y Z** | | | **#2**  **X Y Z** | | | **#1** | **#2** |
| **1** | -32.2 | -27.4 | 61.3 | -34.9 | -26.8 | 56.5 | BA4aL | BA4pL |
| **2** | -4.5 | -35.4 | 0.5 | -35.1 | -24.1 | 49.3 | Brainstem | BA3aL |
| **3** | 13.2 | -56.2 | -25.9 | 18.3 | -57.4 | -25.3 | Cerebellum R | Cerebellum R |
| **4** | -44.7 | -31.9 | 58.9 | -35.7 | -23.8 | 55.1 | BA1L | BA4pL |
| **5** | -36.4 | -17.8 | 41.2 | -36.3 | -17.5 | 44.5 | BA4pL | BA4pL |
| **6** | -43.7 | -25.2 | 50.2 | -39.5 | -26.3 | 61.2 | BA2L | BA4aL |
| **7** | 15.8 | -53.4 | -20.5 | 16.1 | -54.8 | -17.9 | Cerebellum R | Cerebellum R |
| **8** | -34.2 | -9.6 | 64.6 | -24.6 | -38.5 | 70.2 | BA6L | BA1L |
| **9** | -19.9 | 3.3 | 64.3 | -12.1 | 2.7 | 63.4 | BA6L | BA6L |
| **10** | -25.4 | -46.4 | 42.5 | -40.0 | -22.3 | 48.2 | h1P1L | BA3b L |

**Additional file part 2. Table 2.** Individual data and coefficients of variation (CV) in healthy subjects for the mean BOLD activities expressed as percent increase compared with baseline. Data are presented for Brodmann areas BA4a, BA4p, and BA6 according to the Jülich probability atlas for the left hemisphere (L1 &. L2), right hemisphere (R1&.R2), and left-to-right ratios ((L-R)/R*100; %). * p<0.05, comparisons between session 1 & 2.

| **Subj. #** | **BA4a** | | **BA4p** | | **BA6** | |
| --- | --- | --- | --- | --- | --- | --- |
|  | **Bold mean** | | **Bold mean** | | **Bold mean** | |
|  | **L1;L2** | **R1;R2** | **L1;L2** | **R1;R2** | **L1;L2** | **R1;R2** |
| **1** | 1.6;1.2 | 0.6;0.7 | 1.3;1.2 | 0.5;0.3 | 1.3;1.2 | 0.9;0.7 |
| **2** | 0.4;0.8 | 0.3;0.2 | 0.4;0.7 | 0.2;0.1 | 0.3;0.7 | 0.2;0.3 |
| **3** | 0.7**;**1.3 | 0.6**;**0.8 | 0.7;1.3 | 0.4;0.6 | 0.6;1.1 | 0.5;0.8 |
| **4** | 1.0**;**1.0 | 0.5**;**0.4 | 0.8;0.8 | 0.4;0.5 | 1.2;1.1 | 0.8;0.8 |
| **5** | 1.2**;**1.2 | 0.7**;**0.5 | 1.1;1.1 | 0.6;0.5 | 1.1;1.0 | 0.8;0.6 |
| **6** | 1.4**;**0.9 | 0.6**;**0.4 | 1.2;0.7 | 0.5;0.4 | 1.1;0.7 | 0.5;0.3 |
| **7** | 1.7**;**0.9 | 1.0**;**0.6 | 1.2;0.6 | 0.7;0.3 | 1.1;0.8 | 0.8;0.5 |
| **8** | 1.6**;**1.4 | 1.1**;**0.7 | 1.3;1.1 | 0.8;0.4 | 1.8;1.3 | 1.2;0.6 |
| **9** | 1.3**;**0.7 | 0.9**;**0.3 | 1.2;0.5 | 0.7;0.2 | 1.6;0.8 | 0.9;0.5 |
| **10** | 0.7**;**0.9 | 0.5**;**0.4 | 0.4;0.7 | 0.4;0.3 | 0.8;0.8 | 0.5;0.5 |
| **Mean** | 1.2**;**1.1 | 0.7**;**0.5* | 0.9;0.9 | 0.5;0.4* | 1.1;0.9 | 0.7;0.6 |
| **SD** | 0.4**;**0.3 | 0.3**;**0.2 | 0.4;0.3 | 0.2;0.1 | 0.4;0.2 | 0.3;0.2 |
| **CV** | 28 | 35 | 32 | 39 | 27 | 32 |
| **Left-to-Right ratio** | | | | | | |
| **Session** | 1 | 2 | 1 | 2 | 1 | 2 |
|  | 79 | 130 | 85 | 155* | 61 | 76 |

**Additional file part 2. Table 3.** Individual data and coefficients of variation (CV) in healthy subjects for the maximum BOLD activities expressed as percent increase compared with baseline. Data are presented for Brodmann areas BA4a, BA4p, and BA6 according to the Jülich probability atlas for the left hemisphere (L1 & L2), right hemisphere (R1& R2), and left -to-right ratios ((L-R)/R*100; %).

| **Subj. #** | **BA4a** | | **BA4p** | | **BA6** | |
| --- | --- | --- | --- | --- | --- | --- |
|  | **Bold max** | | **Bold max** | | **Bold max** | |
|  | **L1;L2** | **R1;R2** | **L1;L2** | **R1;R2** | **L1;L2** | **R1;R2** |
| **1** | 13.6**;**12.5 | 4.3**;**7.0 | 13.6;12.5 | 2.7;4.3 | 13.6;14.1 | 5.7;6.5 |
| **2** | 4.4;5.0 | 2.1;2.0 | 4.4;3.9 | 1.6;1.0 | 2.7;5.0 | 1.6;2.3 |
| **3** | 4.9**;**10.1 | 2.7**;**4.6 | 4.9;10.1 | 2.7;3.8 | 4.9;10.1 | 3.4;5.1 |
| **4** | 6.8**;**5.1 | 3.1**;**1.8 | 5.2;5.1 | 2.9;3.4 | 10.6; 7.7 | 6.2;7.7 |
| **5** | 10.8**;**8.2 | 4.5**;**4.6 | 6.2;5.4 | 4.2;3.4 | 10.8; 8.2 | 5.2;4.7 |
| **6** | 17.5**;**6.5 | 5.4**;**2.3 | 10.4;4.8 | 3.3;2.1 | 17.5; 6.5 | 2.6;2.3 |
| **7** | 16.6**;**8.9 | 9.9**;**5.5 | 11.5;5.3 | 5.4;3.7 | 11.5;8.2 | 9.9;5.5 |
| **8** | 15.6**;**11.7 | 13.5**;**5.7 | 10.3;11.7 | 9.2;3.7 | 26.4;18.7 | 14.2;5.7 |
| **9** | 8.9**;**6.4 | 8.0**;**1.2 | 8.8;3.0 | 6.1;1.2 | 22.3;11.9 | 8.2; 4.6 |
| **10** | 6.9**;**5.8 | 3.5**;**2.0 | 5.5;5.5 | 3.5;2.0 | 6.9; 5.8 | 3.5; 2.5 |
| **Mean** | 10.6**;**8.0 | 5.7**;**3.7 | 8.1;6.7 | 4.2;2.9 | 12.7;9.6 | 6.1;4.7 |
| **SD** | 5.0**;**2.7 | 3.7**;**2.0 | 3.3;3.4 | 2.2;1.2 | 7.5;4.2 | 3.8;1.8 |
| **CV** | 37 | 59 | 35 | 52 | 37 | 44 |
| **Left-to-Right ratio** | | | | | | |
| **Session** | 1 | 2 | 1 | 2 | 1 | 2 |
|  | 108 | 160 | 122 | 149 | 134 | 116 |

**Additional file part 2.** **Table 4.** fMRI changes in session #1, 2 and 3 in one healthy individual (#1) performing a motor task consisting of 30 s active finger extension-flexion of the right hand in the full range of motion at a frequency freely chosen (#1 and 2) and metronome guided (#3). The 3-dimensional (3-D) distance between the two voxels with peak activities (#1 vs. #2) is shown with session #1 as reference. In session #3 fMRI changes at metronome guided frequencies of 0.25, 0.5, and 1 Hz are presented. The 3-D distance between the voxels with peak activities is shown with 0.25Hz as reference. Max BOLD values and voxel coordinates within specific Brodmann areas according to the Jülich probability atlas.

| **Session** | **Frequency**  **[Hz]** | **BOLD**  **[Z-max]** | **Voxel coordinates**  **X Y Z** | | | **Jülich area** | **3-D distance [mm]** | |
| --- | --- | --- | --- | --- | --- | --- | --- | --- |
|  | | | | | | | | |
| # 1 | 0.42 | 15.5 | -32.2 | -27.4 | 61.3 | BA4aL | | reference |
| # 2 | 0.71 | 9.8 | -34.9 | -26.8 | 56.5 | BA4pL | | 5.5 |
|  | | | | | | | | |
| # 3 | 0.25 | 11.1 | -22.2 | -29.4 | 68.9 | BA6L | | reference |
|  | 0.5 | 11.3 | -26.4 | -29.6 | 68.4 | BA6L | | 4.23 |
|  | 1.0 | 12.7 | -9.64 | -27.5 | 69.9 | BA4aL | | 12.74 |
